# Sexual Activity, Function, and Satisfaction in Reproductive-Aged Females Living with Chronic Kidney Disease

**DOI:** 10.3390/healthcare12171728

**Published:** 2024-08-30

**Authors:** Kathryn S. Corbett, Danica H. Chang, Victoria J. Riehl-Tonn, Sofia B. Ahmed, Neha Rao, Fareed Kamar, Sandra M. Dumanski

**Affiliations:** 1 Department of Medicine, Cumming School of Medicine, University of Calgary, 3330 Hospital Drive NW, Calgary, AB T2N 4N1, Canada; kathryn.corbett@albertahealthservices.ca (K.S.C.); danica2@ualberta.ca (D.H.C.); victoria.riehltonn@ucalgary.ca (V.J.R.-T.); sofia.ahmed@albertahealthservices.ca (S.B.A.); neha.rao@ucalgary.ca (N.R.); fareed.kamar@albertahealthservices.ca (F.K.); 2 Libin Cardiovascular Institute, 3330 Hospital Drive NW, Calgary, AB T2N 4N1, Canada; 3 O’Brien Institute for Public Health, Cumming School of Medicine, University of Calgary, 3330 Hospital Drive NW, Calgary, AB T2N 4N1, Canada; 4 Department of Medicine, Faculty of Medicine and Dentistry, University of Alberta, 11405 87 Ave NW, Edmonton, AB T6G 2R3, Canada

**Keywords:** sexual health, sexual function, sexual activity, sexual satisfaction, chronic kidney disease, women’s health, reproduction

## Abstract

Up to 80% of women living with chronic kidney disease (CKD) experience sexual dysfunction, though its link with sexual activity and sexual satisfaction is not well understood. Among older women with CKD treated with hemodialysis, the majority report sexual inactivity, though few describe sexual difficulty and most report high sexual satisfaction. Whether this applies to reproductive-aged females living with CKD is yet unknown. This study aimed to assess the sexual activity, function, and satisfaction of reproductive-aged females living with CKD. Self-identified females aged 18–51 years with CKD were recruited from nephrology clinics in Calgary, Canada. Sexual activity, function, and satisfaction were assessed with a modified version of the Female Sexual Function Index. Fifty-seven participants were recruited (35% CKD without kidney replacement therapy, 44% CKD treated with hemodialysis, 9% CKD treated with peritoneal dialysis, 12% CKD treated with kidney transplant) and nearly half (47%) reported sexual activity. Among sexually active participants, there was a high prevalence of sexual dysfunction (67%) and only 25% of participants reported sexual satisfaction. A strong relationship between sexual function and satisfaction was identified. Reproductive-aged females living with CKD are sexually active, though experience high rates of sexual dysfunction and dissatisfaction. These findings emphasize the importance of recognition and management of sexual dysfunction in this important population.

## 1. Introduction

Chronic kidney disease (CKD) affects more than 1 in 10 individuals on a global scale and the prevalence of CKD differs by sex, affecting females at a higher rate compared to males (12% vs. 10%, respectively) [1,2]. Sexual dysfunction is a very common complication of CKD [3] and an improved understanding of the causes and treatments for important symptoms of CKD, including sexual dysfunction, has been identified as #3 in the top 10 research priorities for individuals living with CKD [4]. Despite this, research related to sexual health and function in individuals living with CKD is sparse, and the majority of the published literature addresses only male sexual dysfunction.

Though most of the literature to date addresses sexual dysfunction in individuals living with CKD treated with kidney replacement therapy (KRT), it is estimated that approximately three-quarters of individuals living with CKD experience some form of sexual dysfunction [5,6]. Among females, sexual dysfunction may manifest as reduced sexual desire, inadequate vaginal lubrication, dyspareunia or genito-pelvic pain, or abnormal or absent orgasm [7]. According to a recent meta-analysis, 74% of women living with CKD treated with KRT report sexual dysfunction measured by validated self-reported assessment tools, with the highest prevalence of sexual dysfunction observed in women living with CKD treated with hemodialysis (HD) (80%), followed by peritoneal dialysis (PD) (67%), and kidney transplant (63%) [5]. Furthermore, women living with CKD without KRT also appear to experience high rates of sexual dysfunction, up to 2× more frequently than age-matched healthy controls [8].

Measurement of sexual dysfunction in females is often complicated and challenging [9]. In clinical research, female sexual dysfunction is commonly measured by the Female Sexual Function Index (FSFI) [10], an instrument that is limited by its reduced ability to differentiate between sexual dysfunction and sexual inactivity, often leading to overestimation of female sexual dysfunction in settings in which it is administered to sexually inactive individuals [11]. In part to address this limitation, a modified version of the FSFI containing additional questions to distinguish between sexual inactivity and sexual dysfunction was developed and administered to women living with CKD [12]. The authors observed that the majority of participants were sexually inactive, but few endorsed sexual difficulties. Furthermore, despite the low rates of sexual activity, the majority of women reported satisfaction with their sex life overall. However, this study focused on a specific subset of individuals living with CKD treated with HD, who were older and predominantly post-menopausal (median age: 64 years [IQR: 56,73]), which may influence the generalizability of the findings.

Therefore, our objective was to determine the prevalence of sexual activity, sexual function, and sexual satisfaction among reproductive-aged females living with CKD, across varying categories of disease.

## 2. Materials and Methods

### 2.1. Study Population

Adult individuals ≤51 years living with CKD who self-identified as females were recruited to participate in this study via convenience sampling from nephrology clinics in Calgary, Alberta, Canada. Age ≤51 years was used to identify reproductive-aged females based on the median age of menopause in Canada [13]. Females living with CKD are unique in that they may experience a “functional menopause” [14], and unlike in the general population, the clinical definition of menopause does not correlate with permanent reproductive senescence in the CKD population. In the absence of an accurate method to identify females capable of reproduction or a consensus definition of permanent reproductive senescence in this population, age was used as a surrogate for reproductive status. Potential study participants were identified by a clinical team member through the following criteria: (1) participant is followed by a nephrologist for an estimated glomerular filtration rate (eGFR) < 60 mL/min/1.73 m^2^ of any cause or for kidney failure treated with KRT; (2) participant is ≤51 years at the time of recruitment, and (3) participant has provided confirmation of female sex, regardless of gender identity, by answering “yes” to the question “were you born with a uterus?”. Individuals living with CKD with and without KRT were eligible to participate and all data was stratified by type of CKD, specifically: (1) CKD without KRT, (2) CKD treated with HD, (3) CKD treated with PD, and (4) CKD treated with kidney transplant. Exclusion criteria included: (1) acute kidney injury or CKD followed by a nephrologist for <3 months’ duration, and (2) inability to provide informed consent. Participation was voluntary and study participants received a CAD $10 gift card for participating, which functioned as an incentive and an acknowledgment of the time and effort that was required of participants to complete the study. For this exploratory study, we aimed to recruit a convenience sample of at least 50 participants distributed between all 4 CKD groups; a number of participants expected to provide meaningful data.

### 2.2. Data Collection

Pre-planned educational in-services on study recruitment were provided to the clinical staff at each of the 9 clinics responsible for participant recruitment (Kidney Care CKD Clinic, Post-Kidney Transplant Clinic, Peritoneal Dialysis Clinic, Home Hemodialysis Clinic, and 5 In-Center Hemodialysis Clinics) in Calgary, Alberta, Canada. Through collaboration with each clinic’s manager(s), educational in-services on recruitment methods were customized to both the clinics’ patient population and operations at each site.

Recruitment of study participants occurred through convenience sampling. First, study recruitment posters were placed in each waiting room and clinic examination room in each of the 9 clinics above. Secondly, study invitation cards were provided to female individuals at the time of presentation between September 2021 and August 2023. Potential participants were identified by clinical team members involved in their care and consent to be contacted by the research team was obtained. Following this, the potential participant was approached by a member of the research team (either a core member of the research team or a dedicated clinic recruiter for that site who had received specific education on the study methods) who provided the potential participant with study information and discussed the purpose of the study, as well as the risks and benefits of study participation.

All questions about the study and consent form were answered by the research team member or directed to the principal investigator (SMD), and informed consent was obtained. In response to reduced recruitment from the Peritoneal Dialysis Clinic related to clinic operations and relatively infrequent in-person visits for these clinic patients, study invitation letters and study packages were alternatively mailed to potential participants’ homes. The potential participant was provided with the option to participate in the study by completing and mailing back the study consent form and study package in a pre-addressed, postage-paid envelope.

After obtaining informed consent, each participant completed a self-administered survey that collected demographic information, as well as information related to their medical history, including a detailed kidney health history and reproductive health history.

### 2.3. Assessment of Sexual Activity, Function, and Satisfaction

Sexual activity, function, and satisfaction for each participant were assessed through a self-administered modified version of the FSFI instrument [12]. The FSFI is a 19-item tool that has been validated for assessment of female sexual dysfunction in the previous 4 weeks in 6 different domains: desire, arousal, lubrication, orgasm, satisfaction, and pain [10]. Based on participant responses to individual items, a total score between 2 and 36 is generated, with a total score <26.55 representing sexual dysfunction. We employed the modified FSFI instrument developed by Mor et al. [12], which contains four additional questions to ascertain whether the individual has been sexually active in the previous 4 weeks, reasons for sexual inactivity (if applicable), whether the individual believes they are experiencing sexual difficulty, and whether the individual would want to learn about possible causes and treatment options for sexual difficulty. Additionally, individuals who report sexual inactivity in the previous 4 weeks are directed not to complete the portions of the FSFI that address arousal, lubrication, orgasm, and pain, to avoid misclassification of sexually inactive patients as having sexual dysfunction. Importantly, sexual activity was defined as any caressing, foreplay, masturbation, or vaginal intercourse within the previous 4 weeks, ensuring that all sexually active participants (whether participating in partnered or unpartnered sexual activity) were included. The additional questions were analyzed separately and did not alter the FSFI score other than ensuring that only sexually active participants were assessed for sexual dysfunction and self-reported sexual difficulty.

### 2.4. Statistical Analysis

Participant characteristics and study outcomes were pooled as well as stratified into four groups by type of CKD, specifically: (1) CKD without KRT, (2) CKD treated with HD, (3) CKD treated with PD, and (4) CKD treated with kidney transplant. Data were presented descriptively and reported as medians (first quartile [Q1], third quartile [Q3]) for continuous data and proportions (percentages [%]) for categorical data. Kruskal–Wallis and Fisher’s exact tests were utilized to determine if differences existed between strata for continuous and categorical variables, respectively. Data were organized using Microsoft Excel version 16.86 (Microsoft Corporation, Washington, DC, USA) and all statistical analyses were performed using STATA version 18.0 (StataCorp, College Station, TX, USA). A significance level for all tests was set at *p* < 0.05.

### 2.5. Reporting of Sex and Gender

Terms for sex (i.e., female) and gender (i.e., woman) have historically been used interchangeably in the literature. To avoid any assumptions, we have used unaltered terminology from each cited study, however, it is likely that some of the summarized literature has reported findings on “women” from samples of biologically female participants.

## 3. Results

### 3.1. Participant Characteristics

Participant characteristics are presented in Table 1. In total, 57 individuals were included in the study. Among the participants, 20 (35%) were classified as CKD without KRT, 25 (44%) as CKD treated with HD, 5 (9%) as CKD treated with PD, and 7 (12%) as CKD treated with kidney transplant. The median age of study participants was 38 years with no significant differences observed between CKD groups (*p* = 0.81) and the majority of participants identified as cisgender women (96%; n = 52). Nine ethnic identities were represented in the study population; the majority of participants identified as White (60%; n = 32), followed by Indigenous or Metis (9%; n = 5), and Southeast Asian (9%; n = 5). The most common cause of CKD was diabetes, reported by 21% of participants. Other common causes of CKD included glomerulonephritis (17%), congenital or hereditary etiologies (11%), and hypertension (8%).

The participants’ reproductive health history revealed that most participants experienced current menstrual cycles (64%; n = 36), though 9% (n = 5) described secondary amenorrhea (defined as an absent menstrual cycle for at least 3 months), and 25% (n = 14) were categorized as post-menopausal (defined as an absent menstrual cycle for at least 12 months). Patterns of menstruation varied significantly between CKD groups and a lower frequency of menstruation was demonstrated in participants living with CKD treated with HD or PD compared to participants living with CKD treated with kidney transplant or without KRT (*p* < 0.01). Among post-menopausal participants, most reported symptoms of menopause (57%; n = 8), though the presence of menopausal symptoms differed significantly between groups, with the highest rates observed in individuals living with CKD treated with HD (*p* = 0.04). Only 8% (n = 1) of post-menopausal participants reported use of hormone replacement therapy despite 50% (n = 7) reporting vasomotor symptoms of menopause. Most participants reported no other concurrent gynecologic diagnoses, though a small proportion reported endometriosis, gynecologic malignancy, or uterine fibroids.

### 3.2. Sexual Activity

**Figure 1:**
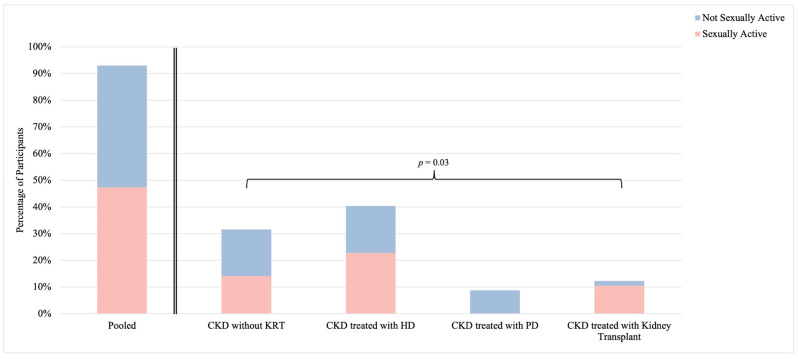
Sexual activity among females living with chronic kidney disease. Stratified by CKD group; *p*-value reports on differences between strata. Abbreviations: CKD, chronic kidney disease; HD, hemodialysis; KRT, kidney replacement therapy; PD, peritoneal dialysis.

The definition of sexual activity included any caressing, foreplay, masturbation, or vaginal intercourse within the previous 4 weeks [12]. Overall, 47% (n = 27) of participants reported sexual activity and sexual activity varied significantly between CKD groups (*p* = 0.03) (Figure 1). Specifically, the highest prevalence of sexual activity was demonstrated among participants with CKD treated with kidney transplant (86%; n = 6), while no participants in the CKD treated with PD group reported sexual activity. The most common reasons for sexual inactivity were lack of a partner (60%; n = 9) and lack of interest in sexual activity (53%; n = 8) (Table S1). Two participants (13%; n = 2) attributed sexual inactivity to self-identified sexual difficulty.

### 3.3. Sexual Function

**Figure 2:**
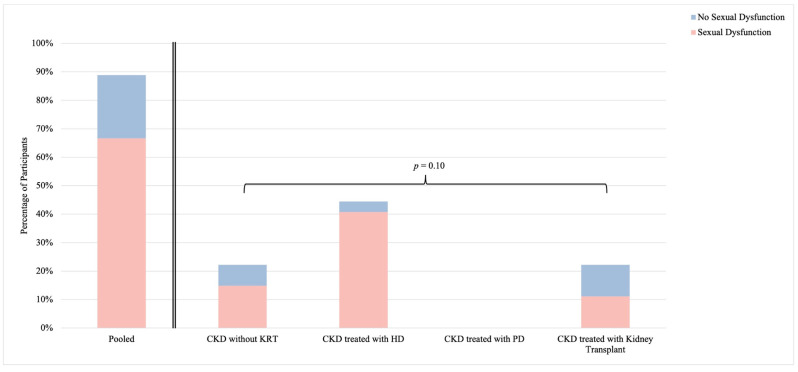
Sexual dysfunction among sexually active females living with chronic kidney disease. Stratified by CKD group; *p*-value reports on differences between strata. Abbreviations: CKD, chronic kidney disease; HD, hemodialysis; KRT, kidney replacement therapy; PD, peritoneal dialysis.

Among the 27 sexually active participants, 24 individuals sufficiently completed the modified FSFI instrument to determine the presence or absence of sexual dysfunction. Overall, 18 sexually active participants (67%) demonstrated an FSFI score indicative of sexual dysfunction (Figure 2). While no statistically significant difference in sexual function was identified between CKD groups (*p* = 0.10), the prevalence of sexual dysfunction was highest among participants in the CKD treated with HD group (85%; n = 11), and lowest among participants in the CKD treated with kidney transplant group (50%; n = 3).

### 3.4. Self-Identified Sexual Difficulty

**Figure 3:**
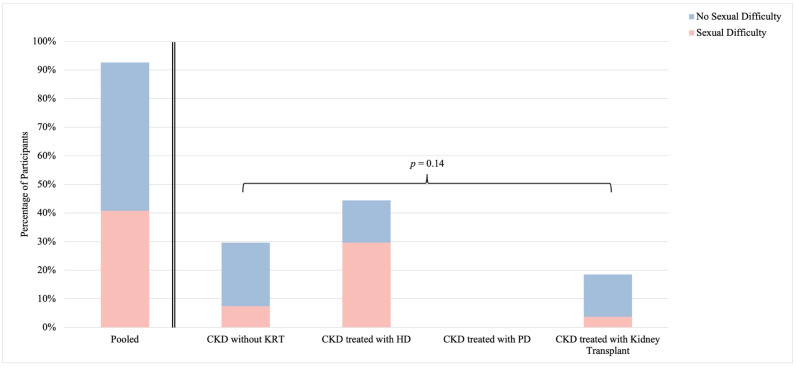
Self-identified sexual difficulty among sexually active females living with chronic kidney disease. Stratified by CKD group; *p*-value reports on differences between strata. Abbreviations: CKD, chronic kidney disease; HD, hemodialysis; KRT, kidney replacement therapy; PD, peritoneal dialysis.

Self-identified sexual difficulty was determined by a yes or no response to the following question: “Would you say that you are experiencing sexual difficulty?”. Among sexually active participants, 41% (n = 11) described sexual difficulty (Figure 3). Similar to sexual dysfunction, the prevalence of self-identified sexual difficulty was highest in participants living with CKD treated with HD (67%; n = 8) and lowest in participants living with CKD treated with a kidney transplant (20%; n = 1), however, this was not significant (*p* = 0.14). Overall, the prevalence of self-identified sexual difficulty was not significantly lower than the prevalence of FSFI-defined sexual dysfunction in sexually active individuals (p=0.10) and although not significant, a trend towards concordance between self-identified sexual difficulty and sexual dysfunction was identified (*p* = 0.15) (Figure S1). Among sexually active participants reporting sexual difficulty, 36% (n = 4) of participants were interested in learning about the potential causes and 55% (n = 6) were interested in learning about possible treatment options (Table S2).

### 3.5. Sexual Satisfaction

**Figure 4:**
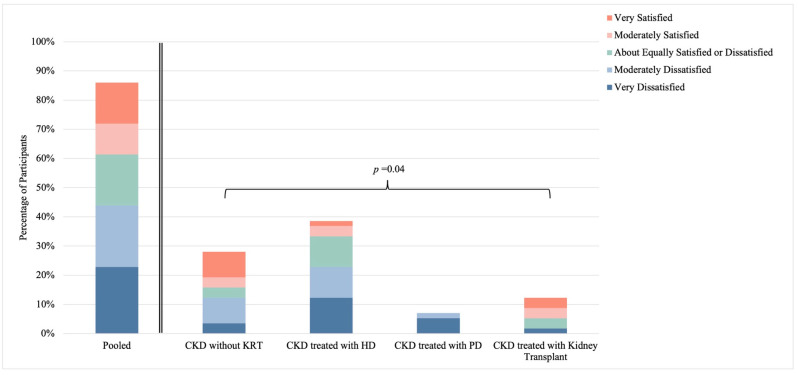
Sexual satisfaction among females living with chronic kidney disease. Stratified by CKD group; *p*-value reports on differences between strata. Abbreviations: CKD, chronic kidney disease; HD, hemodialysis; KRT, kidney replacement therapy; PD, peritoneal dialysis.

**Figure 5:**
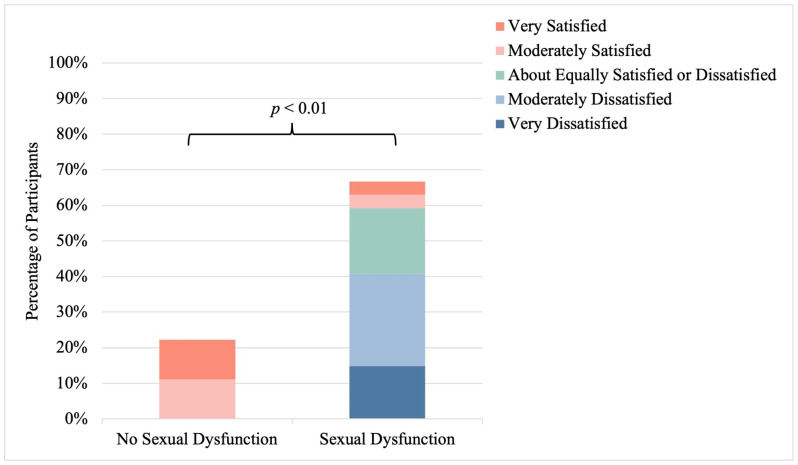
Sexual satisfaction among sexually active females living with chronic kidney disease. Stratified by sexual dysfunction; *p*-value reports on differences between strata.

Overall, only 25% (n = 14) of participants reported sexual satisfaction, while 44% (n = 25) reported dissatisfaction (Figure 4). Among participants reporting sexual dissatisfaction, 21% (n = 12) were moderately dissatisfied and 23% (n = 13) were very dissatisfied. Significant differences in sexual satisfaction were observed between CKD groups, with a higher prevalence of sexual satisfaction among participants living with CKD treated with kidney transplant and CKD without KRT compared to participants living with CKD treated with HD or PD (*p* = 0.04). Additionally, sexual satisfaction was strongly linked to sexual function in sexually active individuals, with a higher prevalence of sexual satisfaction observed in the absence of sexual dysfunction (100%; n = 6) than in the presence of sexual dysfunction (8%; n = 2) (*p* < 0.01) (Figure 5). No significant relationship between sexual satisfaction and sexual activity was observed (*p* = 0.53) (Figure S2).

## 4. Discussion

This cross-sectional exploratory study sought to examine the sexual activity, function, and satisfaction of reproductive-aged females living with CKD. The key findings were as follows: (1) nearly half (47%) of reproductive-aged females living with CKD reported sexual activity; (2) among sexually active participants, there was a high prevalence of sexual dysfunction (67%) and self-identified sexual difficulty (41%); (3) few reproductive-aged females living with CKD reported sexual satisfaction (25%). Taken together, these results both highlight the importance of recognition and management of sexual dysfunction in reproductive-aged females living with CKD and advocate for urgent advancement in research and clinical care to improve the overall sexual well-being of this important population.

The prevalence of sexual activity, even in the general population, is difficult to quantify [15]. Previous studies in the general population have demonstrated a wide range of sexual activity rates in middle-aged women, specifically between 50 and 71% [16,17,18]. The prevalence of sexual activity in our study closely reflected the prevalence reported in the general population and nearly half (47%) of participants reported sexual activity in the previous 4 weeks. Interestingly, there was a significant difference in sexual activity between CKD groups—the prevalence of sexual activity was highest in participants living with CKD treated with kidney transplant (85%) and lowest in participants living with CKD treated with PD (0%), while CKD without KRT and CKD treated with HD reported a sexual activity prevalence of 44% and 57%, respectively. To our knowledge, this is the first study to assess the prevalence of sexual activity in females living with CKD without KRT and CKD treated with PD, but in line with our results, previous studies in the CKD population have observed sexual activity rates of 20–40% in women living with CKD treated with HD and up to 88% in women living with CKD treated with kidney transplant [12,19,20]. The younger age of individuals included in our study likely played an important role in the higher prevalence of sexual activity, as rates of sexual activity in women appear to decline alongside increasing age [17,21].

There are numerous disease-related factors that influence the sexual health of females living with CKD, including CKD-associated fatigue, medication side effects, medicalization of intimate spaces, and comorbid conditions such as depression [22]. Additionally, CKD has an important impact on body image and CKD-associated body image concerns may include dialysis-related features such as fistulas and dialysis catheters [23]. Body image is an important factor influencing sexual activity and function, particularly in women [24,25], and may have an impact on the prevalence of sexual activity in reproductive-aged females living with CKD. Specifically, in our study, nearly two-thirds of sexually inactive participants reported that they were sexually inactive due to a lack of a partner, and a healthy body image has been identified as critically important for the development of intimate partnerships for young individuals living with CKD [25]. Interestingly, though our sample size was limited, participants living with CKD treated with HD or PD—treatments which may have the largest potential for body image impact—reported lack of a partner as a reason for sexual inactivity more commonly than participants living with CKD treated with kidney transplant or CKD without KRT.

While the prevalence of female sexual dysfunction in the general population remains unclear, it has been estimated as high as 40% [26,27]. However, this likely overestimates the true prevalence of sexual dysfunction in reproductive-aged females, as sexual function appears to decline with age [28], and most studies include both pre- and post-menopausal populations. The prevalence of sexual dysfunction has been estimated to be much higher in the CKD population, specifically between 50 and 80% depending on the category of CKD [5,8,29]. Our study observed similar rates of sexual dysfunction—67% overall—with numeric differences between CKD groups, though this did not reach statistical significance. Concordant with previously published studies, we observed the highest prevalence of sexual dysfunction in females living with CKD treated with HD (85%) and the lowest in females living with CKD treated with kidney transplant (50%). Further, our results have the potential to underestimate the prevalence of sexual dysfunction across all CKD groups. Specifically, in sexually inactive participants, 53% reported that a lack of interest in sex was a reason for sexual inactivity. While this response may represent reduced sexual desire that does not cause distress in some participants, it is likely that it represents a sexual interest disorder in other participants, which itself is a manifestation of sexual dysfunction [7].

The documented differences in sexual function between categories of CKD may be in part related to CKD-associated disruptions in hypothalamic–pituitary–ovarian (HPO) axis function, which appear to escalate alongside the progression of CKD [3,14]. The resultant hormonal abnormalities, such as reduced estradiol and elevated prolactin, likely have an important influence on sexual function. Following kidney transplantation, marked improvement in HPO axis function and movement towards normalization of sex hormones has been demonstrated [3,14]. In females, this is often associated with improvement in both menstruation and fertility [14,30]. It is therefore not surprising that in our study, participants living with CKD treated with kidney transplant experienced the highest prevalence of sexual activity and lowest prevalence of sexual dysfunction, likely related in part to increased relative function of the HPO axis. On the other hand, participants living with CKD treated with HD reported the highest prevalence of sexual dysfunction and it was altogether not possible to determine the prevalence of sexual dysfunction among participants living with CKD treated with PD (as all participants in this group were sexually inactive). In line with our expectations, both of these groups demonstrated evidence suggestive of the most severe HPO axis dysfunction, with a significantly lower prevalence of current menstruation (40% in HD, 50% in PD) and a higher prevalence of secondary amenorrhea and menopause—specifically, more than half of reproductive-aged participants living with CKD treated with HD reported absent menstruation. Additionally, this group was more likely to have symptoms of menopause (both genitourinary and vasomotor) and despite a high rate of vasomotor symptoms of menopause, only one participant was treated with hormone replacement therapy. The high prevalence of sexual dysfunction and inactivity in CKD treated with HD, alongside the additional evidence of HPO impairment, highlights a critical need for further study to aid in understanding the clinical effects of HPO axis disruption in CKD, alongside safe and effective treatment strategies for hormonal abnormalities and menopause in females living with CKD.

For females living with CKD, the most common hormonal disturbances identified with CKD-associated HPO axis dysfunction with the potential to impact sexual function include elevated prolactin and reduced estradiol levels [3,14]. While the mechanism and nature of the relationship are not well understood, individuals with hyperprolactinemia report a graded reduction in sexual desire as well as other sexual dysfunction when compared to individuals with normal prolactin levels [31]. In other populations, dopamine agonists have proven helpful in the reduction of prolactin and the improvement of sexual desire and function [32], and may also provide some benefit for the management of sexual dysfunction in females living with CKD. Reductions in estradiol levels, on the other hand, can contribute to vaginal atrophy, dyspareunia, and resultant broad sexual dysfunction [3]. However, treatment with hormone replacement therapy (and specifically topical estrogen) may result in substantial improvement of sexual function in females living with CKD [33]. Although treatment of female sexual dysfunction among individuals living with CKD should be multi-faceted, targeted hormonal therapies may provide significant benefits.

Though not significant, the prevalence of self-identified sexual difficulty among sexually active participants (41%) was lower than the prevalence of FSFI-defined sexual dysfunction (67%) and followed the same trend toward differences between CKD groups. These results were similar to the results reported by Mor and colleagues, which demonstrated that self-identified sexual difficulty (11%) was observed less commonly compared to FSFI-defined sexual dysfunction (43%) [12]. It is likely that this incongruence represents the fact that not all sexual dysfunction results in personal distress, and this lack of associated distress may influence an individual’s awareness and perception of sexual difficulty [7].

Overall, a very low prevalence of sexual satisfaction was demonstrated in this study and only 25% of participants reported satisfaction with their overall sex life. It is recognized that overall health has a large influence on sexual satisfaction [34], therefore reduced sexual satisfaction in this population with chronic disease is not entirely unexpected. Similar to the patterns in sexual activity and function, participants living with CKD treated with kidney transplants had the highest prevalence of sexual satisfaction (57%). Of particular interest in our study, while no relationship between sexual activity and sexual satisfaction was observed, a strong relationship between sexual function and sexual satisfaction was identified. Specifically, in our study, of all sexually active participants without sexual dysfunction, all reported concurrent sexual satisfaction. These results suggest that sexual dysfunction may cause personal distress in reproductive-aged females living with CKD and highlight the critical significance of sexual function in overall sexual satisfaction, further promoting the importance of improved sexual health care for females living with CKD. In fact, sexual satisfaction is a fundamental component of overall wellbeing and leads to improved quality of life [35].

The importance of assessment and management of sexual dysfunction in females living with CKD is highlighted by our study results that reveal that more than half of individuals reporting self-identified sexual difficulty were interested in learning about possible treatment options. The desire for effective management of sexual dysfunction remains an unmet need for individuals living with CKD, and despite a strong desire for information on sexual health from their CKD care providers, the vast majority do not receive any sexual health care in the context of their CKD [36,37]. This is likely more pronounced for female sexual function as compared to male sexual function, as nephrologists report low confidence in the management of women’s health issues in CKD, overall [38,39]. Identified barriers to nephrologists’ provision of sexual health care included patients not raising the issue spontaneously, insufficient time, and lack of knowledge. Our study findings assist in highlighting the urgent need for educational interventions to increase nephrologists’ knowledge and comfort related to the diagnosis and management of sexual dysfunction in females with CKD. As an important priority for individuals living with CKD [4], it is critical for nephrology clinical care providers to include sexual health and function as part of their clinical assessment and management plans. Identification of sexual dysfunction, including a history of the type of sexual dysfunction, onset, and severity, as well as a full reproductive health history, is a key component to further workup and management. Management strategies for sexual dysfunction may include lifestyle and psychosocial counseling, management of comorbidities or modification of culprit medications, optimization of CKD complication management or escalation of dialysis dose and assessment for kidney transplantation, or finally, hormonal therapies [40].

This study has several notable strengths. It is the first study to our knowledge to investigate the prevalence of sexual activity, function, and satisfaction specifically among reproductive-aged females living with CKD, and one of the first studies to examine the prevalence of sexual satisfaction and activity in the female CKD population. Additionally, our study population demonstrated notable diversity in terms of age, and ethnicity, as well as both cause and classification of CKD, thereby strengthening the generalizability of our results. Finally, the use of a self-administered and standardized tool was a strength in terms of obtaining consistent data; however, we recognize that despite clear face validity, this tool has not been formally validated in the CKD population. The use of an unvalidated data collection tool may have impaired our accuracy in measuring what we intended to measure, however, the additional questions added to the modified FSFI utilized did not impact the presentation or scoring of the original FSFI tool that was fully incorporated into the modified version, which is reassuring. Future validation studies of this modified FSFI would be beneficial to assess both the criterion and construct validity of the modified tool. While our study provides valuable insights, it is important to acknowledge several important limitations. First, the study sample was derived from a single geographic location (Calgary, Canada) and may not be generalizable to a wider patient population. To appropriately capture the diversity of the population of reproductive-aged females living with CKD, future studies should endeavor to recruit a large sample size from multiple centers and geographic locations. Second, the cross-sectional nature of this study limited our ability to examine trends over time, and the reduced participation of females living with CKD treated with PD or kidney transplants reduced the statistical power, thereby decreasing the likelihood of detecting and evaluating true differences between groups. As an example, since all participants living with CKD treated with PD were sexually inactive, we were not able to assess sexual function in this group. It is our recommendation that future studies that assess sexual function in this important population are designed to assess longitudinal data to mitigate this important limitation. Third, our sample size was limited, which may impede the generalizability of this work. However, we are confident that this work will contribute to foundational knowledge that will shape future research to develop a more comprehensive understanding of sexual health in females living with CKD. Additionally, recruitment of participants through convenience sampling may have resulted in selection bias and the lack of a control group limited our ability to directly compare to the general population. Nonetheless, this exploratory study is hypothesis-generating and may stimulate larger-scale studies in this important area of clinical science. Finally, we did not collect data on concurrent depression or pharmacologic management of depression in participants, which is an important limitation given that depression +/− its pharmacologic treatments have been independently associated with sexual dysfunction. However, in light of the high prevalence of concurrent depression in the CKD population [41], we believe that this work still provides important value in its accurate description of the prevalence of sexual activity, function, and satisfaction in the female CKD population, recognizing that multiple factors (including depression) are likely to contribute to participants’ overall sexual wellness.

## 5. Conclusions

In summary, this study demonstrated that reproductive-aged females living with CKD have a high prevalence of sexual activity, though also experience notable sexual dysfunction and sexual dissatisfaction. Furthermore, there is a strong relationship between sexual function and sexual satisfaction, which suggests that improvement in sexual function may result in improved sexual satisfaction, an important indicator of overall wellness. Finally, although nephrology patient–provider conversations about sexual health are rare, our study suggests that most sexually active reproductive-aged females living with CKD that report sexual difficulty are interested in learning about possible treatment options for sexual dysfunction.

Overall, despite sexual dysfunction being a patient-identified research priority and accumulating evidence that it is a significant issue among females living with CKD leading to personal distress and sexual dissatisfaction, there is limited research regarding its optimal assessment and management. Furthermore, as a result, there are no evidence-based guidelines to guide nephrologists in the management of female sexual dysfunction in CKD. We are hopeful that this exploratory cross-sectional study will provide an important contribution to this significant gap in knowledge and understanding, and inform larger, multicenter, and more comprehensive prospective studies that will further evaluate the causes and consequences of sexual dysfunction in the female CKD population. Additionally, large well-designed studies are imperative to identify potential treatment targets and interventions that will contribute to targeted and personalized management strategies. Finally, qualitative assessment of the perspectives of reproductive-aged females living with CKD, as well as nephrology care providers, is critical to understanding both barriers and facilitators for effective sexual health care. In summary, further research is urgently needed to identify and operationalize efficacious diagnostic and treatment strategies for sexual dysfunction in females living with CKD.

## Figures and Tables

**Table 1:** Participant characteristics.

	Pooled (n = 57)	CKD without KRT (n = 20)	CKD Treated with HD (n = 25)	CKD Treated with PD (n = 5)	CKD Treated with Kidney Transplant (n = 7)	*p*-Value
**Age (years)**	38 (32, 47) ^a^	40 (32, 47)	37 (32, 47) ^b^	35 (25, 40)	37 (36, 41)	0.81
**Self-identified Ethnicity n (%) ^c^**						0.67
Black	3 (6)	1 (6)	1 (4)	1 (20)	0 (0)	
Caribbean	1 (2)	0 (0)	1 (4)	0 (0)	0 (0)	
East Asian	2 (4)	2 (11)	0 (0)	0 (0)	0 (0)	
Filipinx	1 (2)	1 (6)	0 (0)	0 (0)	0 (0)	
Indigenous or Metis	5 (9)	1 (6)	3 (13)	1 (20)	0 (0)	
Latinx	1 (2)	0 (0)	1 (4)	0 (0)	0 (0)	
South Asian	4 (6)	3 (17)	0 (0)	0 (0)	1 (14)	
Southeast Asian	5 (9)	2 (11)	2 (9)	0 (0)	1 (14)	
White	32 (60)	8 (44)	16 (70)	3 (60)	5 (71)	
**Gender Identity n (%) ^d^**						0.49
Cisgender woman	52 (96)	19 (95)	22 (100)	5 (100)	6 (86)	
Prefer not to say	2 (4)	1 (5)	0 (0)	0 (0)	1 (14)	
**Cause of CKD n (%) ^e^**						0.87
Acute kidney injury	2 (4)	0 (0)	2 (9)	0 (0)	0 (0)	
Congenital/hereditary	6 (11)	3 (16)	2 (9)	0 (0)	1 (14)	
Diabetes	11 (21)	3 (16)	6 (26)	2 (50)	0 (0)	
Glomerulonephritis	9 (17)	3 (16)	4 (17)	1 (25)	1 (14)	
Hypertension	4 (8)	1 (5)	1 (4)	0 (0)	2 (29)	
Polycystic kidney disease	3 (6)	1 (5)	2 (9)	0 (0)	0 (0)	
Reflux nephropathy	2 (4)	0 (0)	1 (4)	0 (0)	1 (14)	
Unknown/other	16 (30)	8 (42)	5 (22)	1 (25)	2 (29)	
**Stage of CKD (among “CKD without KRT” participants only) n (%) ^f^**						N/A
Stage 3a	5 (28)	5 (28)	-	-	-	
Stage 3b	6 (33)	6 (33)	-	-	-	
Stage 4	2 (11)	2 (11)	-	-	-	
Stage 5	4 (22)	4 (22)	-	-	-	
Unsure	1 (6)	1 (6)	-	-	-	
**Menstruation n (%) ^a^**						**<0.01**
Current menstrual cycles	36 (64)	18 (90)	10 (40)	2 (50)	6 (86)	
Secondary amenorrhea	5 (9)	0 (0)	3 (12)	2 (50)	0 (0)	
Post-menopausal	14 (25)	2 (10)	11 (44)	0 (0)	1 (14)	
Unsure	1 (2)	0 (0)	1 (4)	0 (0)	0 (0)	
**Menopausal Symptoms (among “post-menopausal” participants only) n (%) ^g^**						**0.04**
Genitourinary	6 (43)	0 (0)	6 (55)	0 (0)	0 (0)	
Vasomotor	7 (50)	0 (0)	7 (64)	0 (0)	0 (0)	
None	6 (43)	2 (100)	3 (27)	0 (0)	1 (100)	
**Use of HRT (among “post-menopausal” participants only) n (%) ^h^**						N/A ^i^
Yes	1 (8)	0 (0)	1 (10)	0 (0)	0 (0)	
No	12 (92)	2 (100)	9 (90)	0 (0)	1 (100)	
**Other Gynecologic Diagnosis n (%) ^e^**						0.50
Endometriosis	1 (2)	0 (0)	1 (5)	0 (0)	0 (0)	
Gynecologic malignancy	3 (6)	0 (0)	2 (9)	0 (0)	1 (14)	
Uterine fibroids	2 (4)	2 (10)	0 (0)	0 (0)	0 (0)	
None	47 (89)	18 (90)	19 (86)	4 (100)	6 (86)	

Data are median (first quartile [Q1], third quartile [Q3]) unless otherwise indicated. *p*-Values are result of Kruskal–Wallis or Fisher’s exact tests to determine statistical differences between groups. ^a^ A total of 56 responses obtained and included. ^b^ A total of 24 responses obtained and included. ^c^ A total of 53 responses obtained and included, proportions/percentages may not add up to 100% as participants were able to identify as multiple ethnicities. ^d^ A total of 54 responses obtained and included. ^e^ A total of 53 responses obtained and included. ^f^ A total of 18 responses obtained and included. ^g^ A total of 14 responses obtained and included, proportions/percentages may not add up to 100% as participants may have reported multiple symptoms. ^h^ A total of 13 responses obtained and included. ^i^ Unable to assess differences with only one participant reporting HRT. Abbreviations: CKD, chronic kidney disease; HD, hemodialysis; HRT, hormone replacement therapy; KRT, kidney replacement therapy; PD, peritoneal dialysis.

## Data Availability

Data is contained within the article.

## Supplementary Materials

The following supporting information can be downloaded at: https://www.mdpi.com/article/10.3390/healthcare12171728/s1, Table S1. Reasons for Sexual Inactivity Among Sexually Inactive Participants. Table S2. Interest in Learning Among Sexually Active Participants Reporting Self-Identified Sexual Difficulty. Figure S1. Sexual Difficulty Among Sexually Active Females Living with Chronic Kidney Disease. Stratified by sexual dysfunction; p-value reports on differences between strata. Abbreviations: CKD, Chronic Kidney Disease; HD, Hemodialysis; KRT, Kidney Replacement Therapy; PD, Peritoneal Dialysis. Figure S2. Sexual Satisfaction Among Females Living with Chronic Kidney Disease. Stratified by sexual activity; p-value reports on differences between strata. Abbreviations: CKD, Chronic Kidney Disease; HD, Hemodialysis; KRT, Kidney Replacement Therapy; PD, Peritoneal Dialysis.
