# Supplementary material for: Sexual Activity, Function, and Satisfaction in Reproductive-Aged Females Living with Chronic Kidney Disease
Source: Healthcare (Basel). 2024 Aug 30;12(17):1728. doi: 10.3390/healthcare12171728 (PMC11395406; doi:10.3390/healthcare12171728)
Supplement: Supplementary file 1 [file healthcare-12-01728-s001.zip › healthcare-3144185-supplementary.pdf]

SUPPLEMENTARY MATERIALS

Table S1. Reasons for Sexual Inactivity Among Sexually Inactive Participants.

	Pooled (n = 15)[a]	CKD without KRT (n = 4)	CKD treated with HD (n = 7)	CKD treated with PD (n = 3)	CKD treated with Kidney Transplant (n = 1)
No partner	9 (60)	1 (25)	5 (71)	3 (100)	0 (0)
Not interested in being sexually active	8 (53)	3 (75)	3 (43)	1 (33)	1 (100)
Experiencing sexual difficulty	2 (13)	0 (0)	2 (29)	0 (0)	0 (0)
Partner not interested or able to engage in sexual activity	2 (13)	1 (25)	0 (0)	0 (0)	1 (100)
No energy	1 (7)	1 (25)	0 (0)	0 (0)	0 (0)

Data are presented as n (%).[a]15 responses from 26 sexually inactive participants obtained and included; proportions/percentages may not add up to 100% as participants were able to select multiple reasons. Abbreviations: CKD, Chronic Kidney Disease; HD, Hemodialysis; KRT, Kidney Replacement Therapy; PD, Peritoneal Dialysis.

Table S2. Interest in Learning Among Sexually Active Participants Reporting Self-Identified Sexual Difficulty.

	Pooled (n = 11)	CKD without KRT (n = 2)	CKD treated with HD (n = 8)	CKD treated with PD (n = 0)	CKD treated with Kidney Transplant (n = 1)
Interested in learning about possible causes of sexual difficulty?					
Yes	4 (36)	0 (0)	4 (50)	0 (0)	0 (0)
No	7 (64)	2 (100)	4 (50)	0 (0)	1 (100)
Interested in learning about possible treatment options?					
Yes	6 (55)	1 (50)	5 (63)	0 (0)	0 (0)
No	5 (45)	1 (50)	3 (38)	0 (0)	1 (100)

Data are presented as n (%). Abbreviations: CKD, Chronic Kidney Disease; HD, Hemodialysis; KRT, Kidney Replacement Therapy; PD, Peritoneal Dialysis.

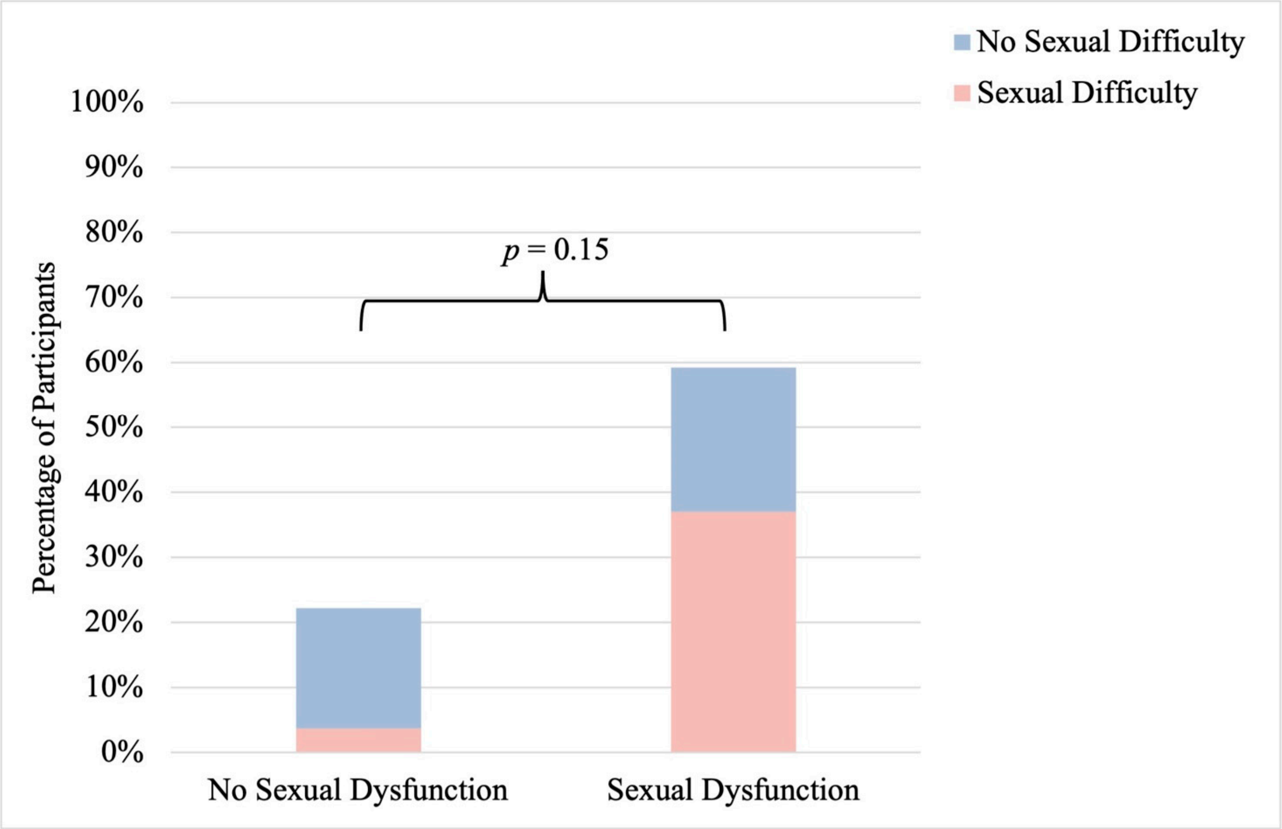

Figure S1. Sexual Difficulty Among Sexually Active Females Living with Chronic Kidney Disease. Stratified by sexual dysfunction; *p*-value reports on differences between strata. Abbreviations: CKD, Chronic Kidney Disease; HD, Hemodialysis; KRT, Kidney Replacement Therapy; PD, Peritoneal Dialysis.

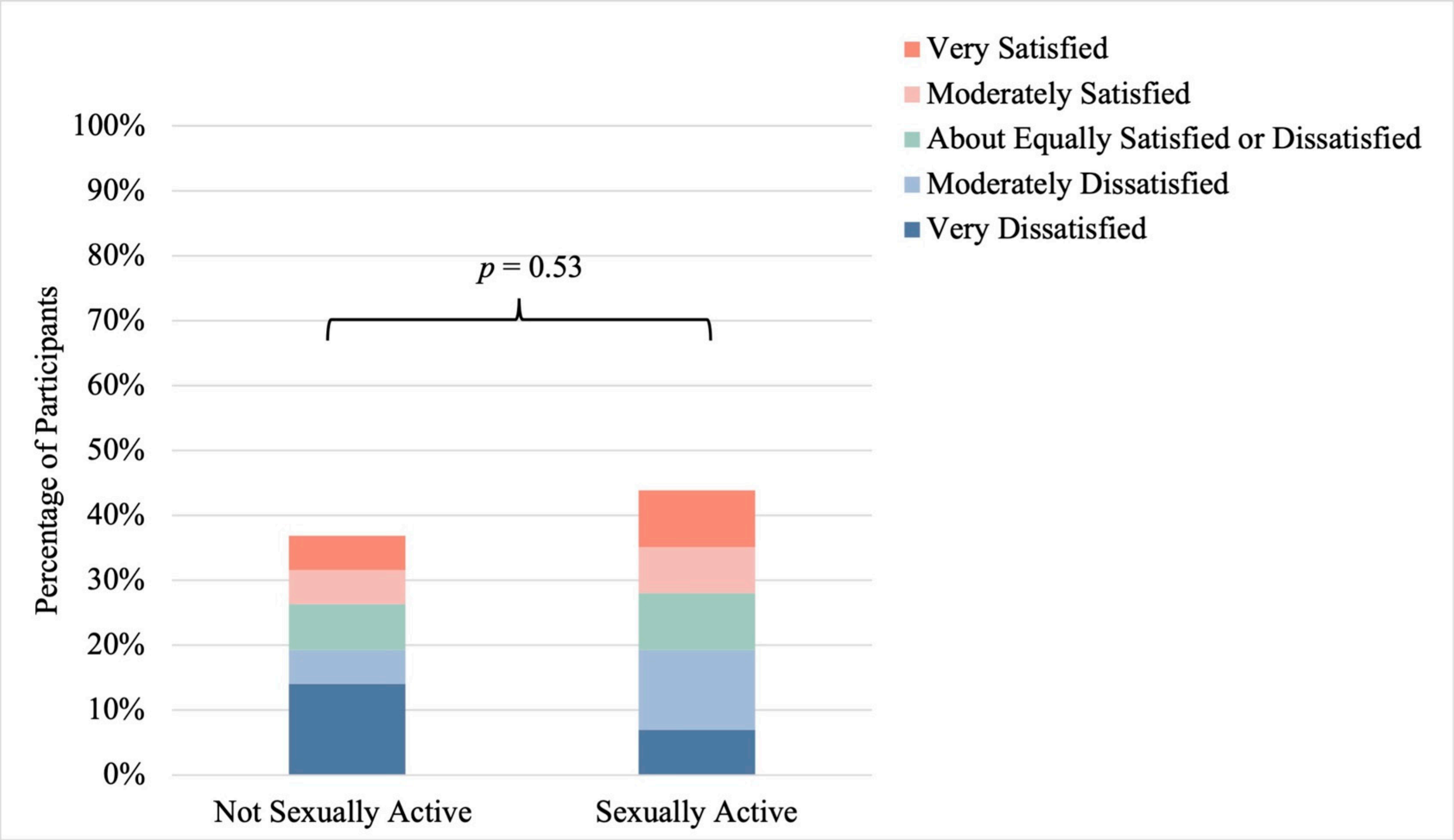

Figure S2. Sexual Satisfaction Among Females Living with Chronic Kidney Disease. Stratified by sexual activity; *p*-value reports on differences between strata. Abbreviations: CKD, Chronic Kidney Disease; HD, Hemodialysis; KRT, Kidney Replacement Therapy; PD, Peritoneal Dialysis.
